# T helper cell mediated-tolerance towards fetal allograft in successful pregnancy

**DOI:** 10.1186/s12948-015-0015-y

**Published:** 2015-06-10

**Authors:** Marie-Pierre Piccinni, Letizia Lombardelli, Federica Logiodice, Ornela Kullolli, Sergio Romagnani, Philippe Le Bouteiller

**Affiliations:** Center of Excellence for Research, Transfer and High Education, DENOTHE of the University of Florence, Florence, Italy; Department of Experimental and Clinical Medicine, University of Florence, Largo Brambilla 3, Florence, 50134 Italy; INSERM UMR1043, CNRS UMR5282, Université de Toulouse III, Centre de Physiopathologie Toulouse-Purpan, Toulouse, 31024 France

**Keywords:** T helper cells, Th1, Th2, Th17, Pregnancy, Abortion, Allograft

## Abstract

Trophoblast HLA-C antigens from paternal origins, which liken the trophoblast to a semiallograft, could be presented by the maternal APCs to the specific maternal CD4+ T helper cells, which could release various cytokines in response to these alloantigens. On the basis of the cytokines produced, these cells can be classified in Th1, Th2 and Th17 cells. Th1 and Th17 cells, known to be responsible for acute allograft rejection, could be involved in miscarriage and Th2 cells together with regulatory CD4+ T cells, known to be involved in allograft tolerance, could be responsible, at least in part, for the success of pregnancy. In this review we focus the role effector CD4+ T cells Th1, Th2 and Th17 cells on the fetal allograft tolerance.

## Introduction

One of the most remarkable aspects of reproductive biology is the fact that a healthy woman can successfully carry a conceptus, liken to an allograft, to full term without rejection.

Although trophoblast does not express HLA class II molecules, it exhibits HLA class I molecules, the polymorphic HLA-C molecules, together with the non-polymorphic HLA-G and HLA-E. For the presence of paternal class I HLA-C molecules on the fetal-derived trophoblast cells, that invade the maternal *decidua basalis*, the conceptus has been considered to be a semi-allograft. After presentation of paternal alloantigens by maternal antigen presenting cells (APCs), the maternal T cells specific for these alloantigens [1], could proliferate and secrete cytokines, promoting the activation of allograft rejection or tolerance mechanisms, respectively responsible for pregnancy failure or fetal survival.

The juxtaposition of the placenta and decidua creates what is refer to as the feto-maternal interface, where placental trophoblasts and uterine lymphocytes most obviously come into contact. It is also possible that the placenta transports cell-free alloantigens from fetal to maternal blood. Thus, even though the anatomy of the pregnant uterus would suggest that the placenta is by far the greatest source of antigens relevant to T cell responses during pregnancy, it remains possible that conceptus-derived antigens are both fetal and placental in origin. The placenta expresses its own set of tissue-specific antigens that should be able to both prime maternal T cells and render the conceptus susceptible to T cell-mediated attack.

The composition and function of the maternal immune cells (8-30%) that populate the decidua are specialized not only to foster placental development and function but also to minimize the chances that the conceptus is attacked as a foreign organ transplant and to combat infections during pregnancy. The immune cell constituents of the fetomaternal interface are during the first trimester pregnancy primarily natural killer (NK) cells (70%), macrophages (20 %) and T cells (10-20%), whereas dendritic cells (DC), B cells and NKT cells are rare [2].

The review will try to cover the functions of CD4+ effector T cells (Th1, Th2 and Th17 cells) from the human feto-maternal interface, given their importance in fetal graft rejection/tolerance. The review will not focus the T reg (CD4+, CD25+, Foxp3+) cells, although they could be responsible for allograft tolerance because it seem that their primary role is to dampen inflammation to prepare implantation [3].

## Review

### Fetal specificity of decidual CD4+ T cells

Understanding the role of decidual T cells in normal and abnormal pregnancy has been limited by an almost complete lack of insight into how many of decidual T cells actually have fetal specificity. In a study to address this issue directly in humans [1], the proportion of activated CD4 + T cells in the decidua of term pregnancies was mildly elevated (10%) if there was a mismatch between maternal and fetal HLA class I (HLA-C) alleles, but not if there was a mismatch in HLA class II molecules. Surprisingly, however, mother/child HLA-C mismatch did not correlate with increases in the proportion of decidual effector CD8+ T cells [1], suggesting that only decidual CD4+ T cells have a fetal specificity. Accordingly, naive maternal CD8+ T cells fail to be primed upon fetal antigen exposure in the secondary lymphoid organs [4].

### Fetal alloantigens presentation to T helper cells

Among the placental antigen presenting cells (APCs), DCs exhibiting high levels of HLA class II molecules, are the most potent activators of naive T cell responses initiating antigen-specific T-cell responses to microbial and transplantation antigens. Upon exposure to pathogens and inflammatory stimuli, DCs migrate via lymphatic vessels to lymphoid organs, where they present antigens together with their HLA class II molecules to naive T cells and direct T cell expansion and polarization.

As CD4^+^ T helper cells can recognize paternal HLA-C molecules only if HLA-C-derived peptides are presented by maternal APCs [1], these results are consistent with the possibility that, T cells in pregnant women recognize conceptus-derived antigens exclusively via indirect allorecognition. Accordingly human early pregnancy decidua DC CD45+, CD40+, HLA-DR+, CD83+, CD1a + cells were often seen in close association with CD3+ T lymphocytes [5]. An early study suggests that maternal CD8+ T cells directly interact with paternal MHC class I molecules expressed by cells of the conceptus [6]. One probable reason for the lack of direct recognition of paternal MHC molecules in mice is the fact that fetal DCs start appearing late in gestation or maternal DCs seem to be trapped within the decidua [7] and cannot reach the uterine draining lymph nodes. As a result of these restrictions, conceptus-derived antigens probably reach the uterine lymph nodes in cell-free form, to be presented by lymph node-resident DCs. The shedding of trophoblast-derived material into maternal blood means that placental antigens also have access to, the spleen and non-uterine lymph nodes, where again they are presented by maternal DCs. After antigen presentation by DCs, effector T cells, which leave the spleen and lymph nodes migrate via the blood to implantation sites, where they might be able to induce fetal demise.

### T cells in pregnancy

Approximately 10–20% of leukocytes in the first-trimester human decidua are CD3 + T cells. About 30–45% of these cells are CD4+ T cells, 45–75% CD8+ T cells and 5% are regulatory T cells (Tregs) with immunosuppressive properties [2].

Consequently to the presentation of paternal HLA-C by maternal APCs the effector CD4+ T helper release various cytokines. On the basis of the profile of cytokines produced, they are classified in T helper (Th)1, Th2 and Th17 cells [8,9]. As these three types of effector T helper cells play a central role in acute allograft rejection and tolerance, we focus the role of these 3 different T subpopulations on fetal allograft tolerance/rejection in human pregnancy. Fetal allograft tolerance by T reg cells is discussed, although T reg cells have also been reported to be responsible for alloantigens tolerance [10], by recent multiple studies in mice suggesting that T reg cells are stimulated by semen antigens, not spermatozoa antigens, thus they play a role in preparing endometrium for implantation dampening uterine inflammation, independently of placental antigen exposure [3].

### Functional activities of Th1, Th2, Th17 CD4+ cells

CD4+ Th1 cells produce interleukin (IL)-2, tumor necrosis factor (TNF)-β and interferon (IFN)-γ and are the main effectors of phagocyte-mediated host defense, which is highly protective against infections sustained by intracellular pathogens. On the other hand, CD4+ Th2 cells, which are mainly responsible for phagocyte-independent host defense against extracellular pathogens, including nematodes, produce IL-4 (which together with IL-10 inhibit several macrophage functions and together with IL-13 produced by Th2 cells, stimulates IgE antibody production) and IL-5 (which promotes growth, differentiation and activation of eosinophils) [8]. An additional subset of CD4 + T helper cells beyond the traditional Th1 and Th2 cells, named Th17, which produce IL-17A, IL17F, IL-21, IL-26 and IL-22 has been identified [9]. The major roles of Th17 is the protection against extracellular bacteria and fungi, on inflammation and on the development of some autoinflammatory diseases [11]. A part of human IL-17A-producing cells were found to also produce interferon (IFN)-γ (they are named Th17/Th1) and both Th17 and Th17/Th1 exhibit plasticity towards Th1 cells in response to IL-12 produced by APCs [11].

### Role of Th1 and Th2 cells on pregnancy development

Putative Th2 and Th17 cells comprise only 5% and 2%, respectively, of first-trimester decidual CD4+ T cells, whereas Th1 cells surprisingly comprise 5–30% of the cells [12].

Proteins and transcripts for intragraft IL-2, IFN-γ and the CTL-specific marker, granzyme B, have been detected in rejecting allografts [13], indicating that some Th1-dependent effector mechanisms play a central role in acute allograft rejection of transplanted tissues [14]. On the other hand, the production of the Th2-type cytokine IL-4 and IL-10, appears to be central for the induction and the maintenance of allograft tolerance [13]. These findings suggested, maybe in a simplistic way, that Th1-type cytokines, that promote allograft rejection, may compromise pregnancy, whereas the Th2-type cytokines inhibiting the Th1 responses, promote allograft tolerance and therefore may improve pregnancy success. Evidence confirmed the role of Th1- and Th2-type cytokines present at fetomaternal interface on the development of pregnancy in mice. First, IL-4, IL-5 and IL-10 were detectable at the fetomaternal interface during all the period of gestation [15,16]. More TNF and IFN-γ and less IL-4 and IL-10 have been found in the uteri of aborting matings of CBA/J female mice with DBA/2 than in CBA/J X Balb/C resulting in normal pregnancy outcome [17]. More importantly, injection of TNF-α, IFN-γ or IL-2 in CBA/J X Balb/C resulted in the increased abortion rates and injection of IL-10 in CBA/J X Balb/C decreased abortion rates [17]. Apparently against the Th1/Th2 paradigm in pregnancy, there are some knock out IL-10 and IL-4-, IL-5-, IL-9- and IL-13-deficient mice which do not affect pregnancy rates and litter size [18]. The ridondancy of cytokines induces sometimes to conclude quickly .

Potential roles of decidual effector CD4+ T cells have emerged from studies on the pathogenesis of Unexplained Recurrent Abortion (URA), with the loss of three or more consecutive pregnancies in the first trimester of pregnancy affects approximately 1 % of the population. In approximately 50% of cases the aetiology is unknown and the proportion of them may be due to immune causes.

Various approaches have been adopted to underline the role of Th1\Th2 in the aetiology of URA\successful pregnancy studing peripheral blood before and at time of miscarriage [19]. Cytokines act locally and the measurement of T cell cytokine at the feto-maternal interface is of greater significance. We found a defect of IL-4 production by both CD4+ and CD8+ T cell clones and a defect of IL-10 by CD4+ T cell clones generated from the decidua of women suffering from URA undergoing a spontaneous abortion in comparison with women undergoing a voluntary abortion [20].

We also found an increased production of Leukemia inhibitory factor (LIF) [21] and macrophage-colony stimulating factor (M-CSF) [22], (endometrial requirements for implantation and embryo development, respectively) by CD4+ T cell clones generated from the decidua of women with a normal pregnancy compared to the decidua of URA [20,23].

The increased production of IL-4, IL-10, LIF and M-CSF at the feto-maternal interface in women with normal pregnancy has not been found at the peripheral blood level, suggesting that this is not an inherent feature of CD4 + T helper cells, but rather a microenvironmentally oriented regulation. Progesterone, which, at concentrations comparable to those present at the feto-maternal interface during pregnancy, is a potent inducer of production of IL-4 [24], but also a potent inducer of LIF and M-CSF production by T cells [20,23] could be at least in part responsible for a Th2 switch at feto-maternal interface and IL-4 produced by the effector CD4+ Th2 cells in turn promote the development of T cells producing M-CSF and LIF important for embryo implantation and development. Very recently, Aisemberg [25] have confirmed these findings, demonstrating in mice that LIF is the potential mediator of the progesterone effect, which is essential for protecting against pregnancy loss. Both IL-4 and IL-10 can inhibit the development and function of Th1 and macrophages cells [26], thus preventing the trophoblast alloantigen rejection. On the other hand, relaxin, a polypeptide hormone predominantly produced by the corpus luteum and decidua during pregnancy, favors the development of T cells producing IFN-γ [27] and may promote an adequate Th1 response to protect the mother against dangerous intracellular pathogens.

Recently, we found that soluble HLA-G5, a soluble isoform of the non classical class I molecule HLA-G, released by trophoblast and embryo, induces IL-4 production by decidual T cells in normal pregnancy. The s HLA-G5-induced IL-4 production by decidual CD4+ T cells is dependant on the level of expression of ILT2, the receptor for HLA-G5, and thus on the state of activation of the decidual CD4+ T cells by trophoblast antigens, but also on the downregulation of ILT2 expression on the decidual macrophages, after they present trophoblast antigens to the decidual CD4+ cells, which unables the macrophages to produce IL-12 (Th1 inducer) in response to HLA-G5 [28]. The role of s HLA-G5, and more importantly, the differential ILT2 expression on uterine CD4+ T cells and macrophages could be critical for recurrent spontaneous abortion.

### Role of decidual Th17 cells on pregnancy development

Th17, has also been described as having a pathogenic role at early stage of allograft rejection [29]. Hence, this early evidence suggests that excessive Th17 activity may promote abortion. Accordingly, Th17 cells has been reported to increase, whereas T reg decrease in decidua of patients with URA compared to healthy pregnant women [30]. Interestingly, it seems that the number of decidual Th17 cells is significantly higher in inevitable abortion cases involving active genital bleeding , but not when abortion cases do not involve active genital bleeding. Therefore, Th17 cells could be involved in the induction of inflammation in the late stage of abortion, but not in the early stage of abortion and thus not in fetal rejection [31].

Very recently, we observed an associated production of IL-4 and IL-17 by a large number of decidual CD4 + T cells (named Th17/Th2 cells) in normal pregnancy and at embryo implantation site, whereas Th17\Th1 cells [11] are prevalent in URA (manuscript submitted). The differentiation of Th17 cells into Th17/Th2 cells already described in allergic disorders [32], seems to be due to soluble HLA-G5 in normal human pregnancy (manuscript submitted). Th17 cells do not show a pathogenetic role for pregnancy, but a beneficial role, when they also produce IL-4. Infection-related immunity during gestation seems to be preferentially directed towards combating extracellular microbial pathogens with Th17-mediated immune responses in the intrauterine environment (both fetal amniotic compartments) [33] suggesting that Th17/Th2 cells could be essential for the success of pregnancy, because they may promote an adequate response to protect the mother against dangerous extracellular pathogens and induce fetoallograft tolerance.

## Conclusion

The success of pregnancy is surely not only the result of a tolerance towards the paternal MHC class I alloantigens, but may also depend from many immune mechanisms, including an adequate response required to protect the mother against dangerous pathogens deleterious for the pregnancy development and an adequate vascularisation of the placenta. Cytokines produced at feto-maternal interface form a complex regulatory network which maintains homeostasis between the maternal immune system and the fetus. If this delicate balance is adversely affected, immunoregulatory mechanisms may be insufficient to restore homeostasis and this may lead to pregnancy failure.

## References

[1] Tilburgs T, Scherjon SA, van der Mast BJ, Versteeg-V D, Voort-Maarschalk M, Roelen DL (2009). Fetal–maternal HLA-C mismatch is associated with decidual T cell activation and induction of functional T regulatory cells. J Reprod Immunol.

[2] Bulmer JN, Williams PJ, Lash GE (2010). Immune cells in the placental bed. Int J Dev Biol.

[3] Robertson SA, Prins JR, Sharkey DJ, Moldenhauer LM (2013). Seminal fluid and the generation of regulatory T cells for embryo implantation. Am J Reprod Immunol.

[4] Erlebacher A, Vencato D, Price KA, Zhang D, Glimcher LH (2007). Constraints in antigen presentation severely restrict T cell recognition of the allogeneic fetus. J Clin Invest.

[5] Kammerer U, Schoppet M, Mc Lellan AD, Kapp M, Huppertz HI, Kämpgen E (2000). Human decidua contains potent immunostimilatory CD83+ dendritic cells. Am J Pathol.

[6] Tafuri A, Alferink J, Moller P, Hammerling GJ, Arnold B (1995). T cell awareness of paternal alloantigens during pregnancy. Science.

[7] Collins MK, Tay CS, Erlebacher A (2009). Dendritic cell entrapment within the pregnant uterus inhibits immune surveillance of the maternal/fetal interface in mice. J Clin Invest.

[8] Romagnani S (1991). Human Th1 and Th2: doubt no more. Immunol Today.

[9] Harrington LE, Hatton RD, Mangan PR, Turner H, Murphy TL, Murphy KM (2005). Interleukin 17-producing CD4+ effector T cells develop via a lineage distinct from the T helper type 1 and 2 lineages. Nat Immunol.

[10] Graca L, Cobolt SP, Waldmann H (2002). Identification of regulatory T cells in tolerated allografts. J Exp Med.

[11] Annunziato F, Cosmi L, Santarlasci V, Maggi L, Liotta F, Mazzinghi B (2007). Phenotypic and functional features of human Th17 cells. J Exp Med.

[12] Mjösberg J, Berg G, Jenmalm MC, Ernerudh J (2010). FOXP3+ regulatory T cells and T helper 1, T helper 2, and T helper 17 cells in human early pregnancy deciduas. Biol Reprod.

[13] Strom TB, Roy-Chaudury R, Manfro R, Zheng XX, Nickerson PW, Wood K (1996). The Th1/Th2 paradigm and allograft response. Curr Opin Immunol.

[14] Burns WR, Wang Y, Tang PC (2005). Recruitment of CXCR3+ and CCR5+ T cells and production of interferon-gamma-inducible chemokines in rejecting human arteries. Am J Transplant.

[15] Lin H, Mosmann TR, Guilbert L, Tuntipopipat S, Wegmann TG (1993). Synthesis of T helper 2-type cytokines at maternal-fetal interface. J Immunol.

[16] Wegmann TG, Lin H, Guilbert L, Mossmann TR (1993). Bidirectional cytokine interactions in the maternal-fetal relationship: is successful pregnancy a Th2 phenomenon?. Immunol Today.

[17] Chaouat G, Assal-Meliani A, Martal J, Raghupathy R, Elliott JF, Mosmann T (1995). Wegmann TG.IL-10 prevents naturally occuring fetal loss in the CBA X DBA/2 mating combination and local defect in IL-10 production in this abortion-prone combination is corrected by in vivo injection of IFN-γ. J Immunol.

[18] Fallon PG, Jolin HE, Smith P, Emson CL, Townsend MJ, Fallon R (2002). IL-4 induces characteristic Th2 responses even in the combined absence of IL-5, IL-9, and IL-13. Immunity.

[19] Raghupathy R, Makhseed M, Azizieh F, Omu A, Gupta M, Farhat R (2000). Cytokine production by maternal lymphocytes during normal human pregnancy and in unexplained recurrent spontaneous abortion. Hum Reprod.

[20] Piccinni MP, Beloni L, Livi C, Maggi E, Scarselli G, Romagnani S (1998). Defective production of both leukemia inhibitory factor and type 2 T-helper cytokines by decidual T cells in unexplained recurrent abortions. Nat Med.

[21] Stewart CL, Kaspar P, Brunet LJ, Bhatt H, Gadi I, Köntgen F (1992). Blastocyst implantation depends on maternal expression of leukemia inhibitory factor. Nature.

[22] Pollard JW, Bartocci A, Arceci R, Orlofsky A, Ladner MB, Stanley ER (1987). Apparent role of the macrophage growth factor, CSF-1, in placental development. Nature.

[23] Piccinni MP, Scaletti C, Vultaggio A, Maggi E, Romagnani S (2001). Defective production of LIF, M-CSF and Th2-type cytokines by T cells at fetomaternal interface is associated with pregnancy loss. J Reprod Immunol.

[24] Piccinni MP, Giudizi MG, Biagiotti R, Beloni L, Giannarini L, Sampognaro S (1995). Progesterone favors the development of human T helper cells producing Th2-type cytokines and promotes both IL-4 production and membrane CD30 expression in established Th1 cells clones. J Immunol.

[25] Aisemberg J, Vercelli CA, Bariani MV, Billi SC, Wolfson ML, Franchi AM (2013). Progesterone is essential for protecting against LPS-induced pregnancy loss. LIF as potential mediator of the anti-inflammatory effect of progesterone). PLoS One.

[26] Bashyam H (2007). Th1/Th2 cross-regulation and the discovery of IL-10. J Exp Med.

[27] Piccinni MP, Bani D, Beloni L, Manuelli C, Mavilia C, Vocioni F (1999). Relaxin favors the development of activated human T cells into Th1-like effectors. Eur J Immunol.

[28] Lombardelli L, Aguerre-Girr M, Logiodice F, Kullolli O, Casart Y, Polgar B (2013). HLA-G5 induces IL-4 secretion critical for successful pregnancy through differential expression of ILT2 receptor on decidual CD4^+^ T cells and macrophages. J Immunol.

[29] Chen H, Wang W, Xie H, Xu X, Wu J, Jiang Z (2009). A pathogenic role of IL- 17 at the early stage of corneal allograft rejection. Transpl Immunol.

[30] Wang WJ, Hao CF, Yi-Lin A, Yin GJ, Bao SH, Qiu LH (2010). Increased prevalence of T helper 17 (Th17) cells in peripheral blood and decidua in unexplained recurrent spontaneous abortion patients. J Reprod Immunol.

[31] Nakashima A, Ito M, Shima T, Bac ND, Hidaka T, Saito S (2010). Accumulation of IL-17-positive cells in decidua of inevitable abortion cases. Am J Reprod Immunol.

[32] Cosmi L, Maggi L, Santarlasci V, Capone M, Cardilicchia E, Frosali F (2010). Identification of a novel subset of human circulating memory CD4(+) T cells that produce both IL-17A and IL-4. J Allergy Clin Immunol.

[33] Witkin SS, Linhares IM, Bongiovanni AM, Herway C, Skupski D (2011). Unique alterations in infection-induced immune activation during pregnancy. BJOG.

